# Chiropractic treatment of scoliosis; a systematic review of the scientific literature

**DOI:** 10.1186/1748-7161-8-S1-O15

**Published:** 2013-06-03

**Authors:** J McAviney

**Affiliations:** 1 Sydney Scoliosis Clinic, Sydney, Australia

## Background

Chiropractors are primary health care practitioners who specialise in the treatment of the spine using manual therapy, in particular spinal manipulation. Chiropractors are ideally placed to diagnose and treat scoliosis through conservative methods. However, in order for the chiropractic profession to assume this role, an understanding of the effectiveness of chiropractic treatment of scoliosis is required.

## Methods

A systematic literature search was performed of the Cochrane Library, Medline, CINAHL and Google Scholar using the search terms, “chiropractic and scoliosis”, “chiropractic treatment and scoliosis”, “spinal manipulation and scoliosis”.

## Results

No high quality RCT’s were found, and no studies reviewed used the Scoliosis Research Society inclusion criteria and 2-year post treatment follow-up. The studies fell into two categories: 1) studies primarily using spinal manipulation, and 2) those using a multimodal rehabilitation based approach. In studies where manipulation was the primary intervention, some case reports reported reduction in the Cobb angle. However, the strongest study from this group was a Cohort time-series trial which studied 42 patients aged 6 to 12 years of age. The intervention was manipulation and heel lifts. They found that “chiropractic adjustments with heel lifts and postural and lifestyle counselling were not effective in reducing the severity of scoliotic curves.”

## Discussion

There is a lack of evidence for chiropractic treatment of scoliosis. From the rehabilitation based treatment studies, most were case series and reported reductions in the Cobb angle at the end of treatment. However, none of these studies reported any follow-up beyond the end of treatment, and most of the patients treated were adults or older adolescents at low risk of scoliosis progression. Available evidence suggests that spinal manipulation does not influence the progression of adolescent scoliosis, and although chiropractic rehabilitation programs may influence Cobb angle, this could be temporary, and only in patients that are not at risk of significant progression.

## Conclusions

Given this evidence, chiropractic specific treatments of spinal manipulation and rehabilitation should not be recommended over treatments that have demonstrated evidence, such as bracing and scoliosis specific rehabilitation programs. If chiropractors wish to play a role in the management of scoliosis, then they should offer evidenced based approaches to scoliosis management.
